# Supplementary material for: Impact of the COVID-19 Pandemic on Lifestyle Behavior and Clinical Care Pathway Management in Type 2 Diabetes: A Retrospective Cross-Sectional Study
Source: Medicina (Kaunas). 2024 Oct 4;60(10):1624. doi: 10.3390/medicina60101624 (PMC11509258; doi:10.3390/medicina60101624)
Supplement: Supplementary file 1 [file medicina-60-01624-s001.zip › Supplementary File S2.pdf]

Supplementary File S2

English version of Pacic questionnaire

No	Lists of questions	Almost	Never	Rarely	Sometimes	Often	Almost always
1	My opinion was asked when we developed a treatment plan.						
2	Various therapeutic alternatives have been proposed to me to reflect on.						
3	I have been asked to talk about any problems I have experienced with the drugs or their effects.						
4	I have been given a written list of things to do to improve my health.						
5	I was able to see that the assistance that was given to me was well organized.						
6	I was shown how what I did to take care of my illness affected my health.						
7	I have been asked to talk about the goals I have set for the treatment of my disease.						
8	I was helped to set specific goals to improve my nutrition or physical activity.						
9	I have been given a copy of my treatment plan.						
10	I was encouraged to attend a specific class or group that can help me cope with my chronic illness.						
11	I have been asked questions, either directly or through a survey, related to my habits for staying healthy.						
12	I was sure that, in recommending the appropriate therapies to me, my doctor or trusted Nurse took my values and traditions into account.						
13	I was helped to develop a feasible treatment plan every day.						
14	I have been helped to make plans for the future so that I can take care of my illness even in times of difficulty.						
15	I have been asked how my chronic illness affects my life.						
16	I have been in contact after a visit to see how the situation was progressing.						
17	I was encouraged to attend local programs that may help me.						
18	I have been referred by a dietician, health educator, or counseling specialist.						
19	It was explained to me that my visits to other specialists (ophthalmologist, surgeon, etc.) have improved the treatment of my disease.						
20	I was asked how my visits to other specialists were proceeding.						
21	I was asked what aspects of my illness I wanted to talk about during the visit.						
22	I have been asked what extent work, family, or social situation relates to my management of the disease.						
23	I have been helped to develop plans to understand how to get support from friends, family or the community in which I live.						

**24**	I have been told how important the things I do to take care of my illness (e.g., physical activity) are important to my health.	
**25**	A goal has been set for me to pursue together with the health personnel who take care of me to manage my disease.	
**26**	I was given a diary to record the progress I make.	

Italian Version of Pacic Questionnaire

	Quasi mai	Raramente	Qualche volta	Spesso	Quasi sempre
1. È stato chiesto il mio parere quando abbiamo elaborato un piano terapeutico.					
2. Mi sono state proposte varie alternative terapeutiche su cui riflettere.					
3. Mi è stato chiesto di parlare di eventuali problemi riscontrati con i farmaci o i loro effetti.					
4. Mi è stato dato un elenco scritto di cose da fare per migliorare la mia salute.					
5. Ho potuto constatare che l'assistenza che mi veniva prestata era ben organizzata.					
6. Mi è stato mostrato come ciò che ho fatto per prendermi cura della mia malattia ha influito sul mio stato di salute.					
7. Mi è stato chiesto di parlare degli obiettivi che mi sono posto/a per il trattamento della mia malattia.					
8. Sono stato/a aiutato/a a definire obiettivi specifici per migliorare la mia alimentazione o la mia attività fisica.					
9. Mi è stata data una copia del mio piano terapeutico.					
10. Sono stato/a incoraggiato/a a frequentare una classe o un gruppo specifico in grado di aiutarmi ad affrontare la mia malattia cronica.					
11. Mi sono state poste delle domande, direttamente o attraverso un sondaggio, relative alle mie abitudini per mantenermi in salute.					
12. Ho avuto la certezza che, nel raccomandarmi le terapie del caso, il mio medico o l'infermiere/a di mia fiducia ha tenuto conto dei miei valori e delle mie tradizioni.					
13. Sono stato/a aiutato/a a elaborare un piano terapeutico fattibile nella mia routine quotidiana.					

14. Sono stato/a aiutato/a elaborare dei piani per il futuro in modo da potermi prendere cura della mia malattia anche in periodi di difficoltà.
15. Mi è stato chiesto in che modo la mia malattia cronica influisce sulla mia vita.
16. Sono stato/a contattato/a dopo una visita per vedere come procedeva la situazione.
17. Sono stato/a incoraggiato/a a frequentare programmi a livello locale che potrebbero aiutarmi.
18. Sono stato/a inviato/a da un dietologo, educatore sanitario o specialista in counseling.
19. Mi è stato spiegato che le visite da me effettuate presso altri specialisti (oftalmologo, chirurgo, ecc.) hanno migliorato il trattamento della mia malattia.
20. Mi è stato chiesto come procedevano le mie visite presso altri specialisti.
21. Mi è stato chiesto di che aspetti della mia malattia volevo parlare nel corso della visita.
22. Mi è stato chiesto in che misura il lavoro, la famiglia o la situazione sociale si relazionano alla mia gestione della malattia.
23. Sono stato/a aiutato/a elaborare dei piani per capire come ricevere supporto dagli amici, dalla famiglia o dalla comunità in cui vivo.
24. Mi è stato detto quanto sono importanti per la mia salute le cose che faccio per prendermi cura della mia malattia (es. attività fisica).
25. Mi è stato posto un obiettivo da perseguire insieme al personale sanitario chi si occupa di me per gestire la mia malattia.
26. Mi è stato dato un diario in cui registrare i progressi che faccio.
