# Supplementary material for: Impact of the COVID-19 Pandemic on Lifestyle Behavior and Clinical Care Pathway Management in Type 2 Diabetes: A Retrospective Cross-Sectional Study
Source: Medicina (Kaunas). 2024 Oct 4;60(10):1624. doi: 10.3390/medicina60101624 (PMC11509258; doi:10.3390/medicina60101624)
Supplement: Supplementary file 1 [file medicina-60-01624-s001.zip › Supplementary File S3.pdf]

Supplementary File S3

English version of Medi-Lite questionnaire

**FRUIT**<br>*1 portion: 150 g*	<1 portion/d<br>0	1-2 portion/d<br>1	>2 portion/d<br>2
**VEGETABLES**<br>*1 portion: 100 g*	<1 portion/d<br>0	1-2.5 portion/d<br>1	>2.5 portion/d<br>2
**LEGUMES**<br>*1 portion: 70 g*	<1 portion/week<br>0	1-2 portion/week<br>1	>2 portion/week<br>2
**CEREALS**<br>*1 portion: 130 g*	<1 portion/d<br>0	1-1,5 portion/d<br>[illegible]	>1.5 portion/d<br>[illegible]
**FISH**<br>*1 portion: 100 g*	<1 portion/week<br>0	[illegible]<br>1	[illegible]<br>2
**MEAT AND MEAT PRODUCTS**<br>*1 portion: 80 g*	<1 portion/d<br>2	1-1.5 portion/d<br>1	>1.5 portion/d<br>0
**DAIRY PRODUCTS**<br>*1 portion: 180 g*	<1 portion/d<br>2	1-1.5 portion/d<br>1	>1.5 portion/d<br>0
**ALCOHOL**<br>*1 Alcohol Unit (AU) = 12 g*	<1 AU/d<br>1	1-2 AU/d<br>2	>2 AU/d<br>0
**OLIVE OIL**	Occasional use<br>0	Frequent use<br>1	Regular use<br>2

Score: 0-18

Total:

Sofi et al., Public Health Nutr 2014

Italian version of Medi-Lite questionnaire

**FRUTTA**<br>*1 porzione: 150 g (esempio: 1 mela, pera, arancia; 3 prugne, mandarini)*	<1 porzione/die	1-2 porzioni/die	>2 porzioni/die
**VERDURA**<br>*1 porzione: 100 g (esempio: 1 piatto di insalata; 2 pomodori; ½ vaschetta di verdura cotta)*	<1 porzione/die	1-2,5 porzioni/die	>2,5 porzioni/die
**LEGUMI**<br>*1 porzione: 70 g (esempio: ½ scatoletta di fagioli o ceci o lenticchie o piselli)*	<1 porzione/sett.	1-2 porzioni/sett.	>2 porzioni/sett.
**CEREALI** (pane, pasta, biscotti etc.)<br>*1 porzione: 130 g (Esempi: 1 porzione pasta: 80 g; 4 biscotti frollini: 50 g)*	<1 porzione/die	1-1,5 porzioni/die	>1,5 porzioni/die
**PESCE** (eccetto molluschi e crostacei)<br>*1 porzione: 100 g*	<1 porzione/sett.	1-2,5 porzioni/sett.	>2,5 porzioni/sett.
**CARNE E SALUMI**<br>*1 porzione: 80 g (Esempi: 1 porzione carne: 100 g 1 porzione salumi: 50 g (esempio: ½ vaschetta prosciutto)*	<1 porzione/die	1-1,5 porzioni/die	>1,5 porzioni/die
**LATTE E LATTICINI**<br>*1 porzione: 180 g (Esempi: 1 tazza di latte: 150 g; 1 yogurt: 125)*	<1 porzione/die	1-1,5 porzioni/die	>1,5 porzioni/die
**ALCOL**<br>*1 U.A. = 1 bicchiere di vino; 1lattina birra*	<1 U.A./die	1-2 U.A./die	>2 U.A./die
**OLIO D'OLIVA**	Occasionalmente	Frequentemente	Regolarmente

Totale:

Adattato da Sofi et al.. Public Health Nutr 2014
