# Supplementary material for: Impact of the COVID-19 Pandemic on Lifestyle Behavior and Clinical Care Pathway Management in Type 2 Diabetes: A Retrospective Cross-Sectional Study
Source: Medicina (Kaunas). 2024 Oct 4;60(10):1624. doi: 10.3390/medicina60101624 (PMC11509258; doi:10.3390/medicina60101624)
Supplement: Supplementary file 1 [file medicina-60-01624-s001.zip › Supplementary File S4.pdf]

Supplementary File S4

Summary Statistics for 5 As Scoring

Overall Summary Statistics

Column	count	mean	std	min	25%	50%	75%	max
**5As Summary Score**	103.00	2.74	0.60	1.40	2.36	2.72	3.10	4.28
**Assess**	103.00	2.35	0.78	1.00	1.80	2.40	2.80	4.40
**Advise**	103.00	2.46	0.79	1.00	1.80	2.40	3.00	4.80
**Agree**	103.00	3.39	0.92	1.00	2.80	3.60	4.00	5.00
**Assist**	103.00	2.88	0.87	1.00	2.20	2.80	3.40	5.00
**Arrange**	103.00	2.62	0.78	1.20	2.00	2.60	3.20	5.00

Gender: M - Summary Statistics

Column	count	mean	std	min	25%	50%	75%	max
**5As Summary Score**	73.00	2.65	0.55	1.40	2.36	2.64	2.96	4.28
**Assess**	73.00	2.26	0.71	1.00	1.80	2.20	2.60	4.40
**Advise**	73.00	2.34	0.72	1.00	1.80	2.40	2.80	3.60
**Agree**	73.00	3.32	0.90	1.00	2.80	3.40	4.00	5.00
**Assist**	73.00	2.79	0.85	1.00	2.20	2.80	3.40	4.60
**Arrange**	73.00	2.53	0.82	1.20	1.80	2.40	3.20	5.00

Gender: F - Summary Statistics

Column	count	mean	std	min	25%	50%	75%	max
**5As Summary Score**	30.00	2.96	0.69	1.60	2.48	2.90	3.35	4.20
**Assess**	30.00	2.58	0.89	1.20	1.80	2.60	3.20	4.20
**Advise**	30.00	2.74	0.91	1.60	1.85	2.60	3.35	4.80
**Agree**	30.00	3.57	0.97	1.00	2.85	3.60	4.20	5.00

**Assist**	30.00	3.10	0.88	1.60	2.45	3.00	3.40	5.00
**Arrange**	30.00	2.83	0.66	1.20	2.60	2.80	3.40	4.20

Educational Qualification: High school - Summary Statistics

Column	count	mean	std	min	25%	50%	75%	max
**5As Summary Score**	30.00	2.63	0.56	1.40	2.37	2.64	2.89	4.08
**Assess**	30.00	2.25	0.77	1.00	1.80	2.40	2.60	4.00
**Advise**	30.00	2.51	0.88	1.00	1.80	2.60	3.15	4.80
**Agree**	30.00	3.25	0.91	1.40	2.85	3.60	3.95	4.60
**Assist**	30.00	2.79	0.87	1.00	2.20	2.80	3.40	4.60
**Arrange**	30.00	2.37	0.63	1.20	1.80	2.20	2.80	3.60

Educational Qualification: Degree - Summary Statistics

Column	count	mean	std	min	25%	50%	75%	max
**5As Summary Score**	5.00	3.28	0.77	2.36	2.68	3.52	3.56	4.28
**Assess**	5.00	3.08	1.16	2.00	2.00	2.80	4.20	4.40
**Advise**	5.00	3.20	0.47	2.40	3.20	3.40	3.40	3.60
**Agree**	5.00	4.08	0.90	2.80	3.60	4.20	4.80	5.00
**Assist**	5.00	3.24	0.90	2.00	2.80	3.40	3.60	4.40
**Arrange**	5.00	2.80	1.08	1.80	1.80	2.60	3.60	4.20

Educational Qualification: Secondary school - Summary Statistics

Column	count	mean	std	min	25%	50%	75%	max
**5As Summary Score**	46.00	2.70	0.65	1.44	2.36	2.72	3.07	4.20
**Assess**	46.00	2.36	0.72	1.20	1.80	2.20	2.80	4.20
**Advise**	46.00	2.33	0.76	1.00	1.80	2.30	2.75	4.20
**Agree**	46.00	3.30	1.02	1.00	2.65	3.40	4.00	5.00

**Assist**	46.00	2.87	0.93	1.20	2.20	2.80	3.40	5.00
**Arrange**	46.00	2.66	0.86	1.20	2.00	2.60	3.20	5.00

Educational Qualification: Primary School - Summary Statistics

Column	count	mean	std	min	25%	50%	75%	max
**5As Summary Score**	22.00	2.83	0.47	2.28	2.42	2.82	3.16	3.88
**Assess**	22.00	2.32	0.77	1.20	1.80	2.20	2.60	4.20
**Advise**	22.00	2.47	0.75	1.60	1.85	2.20	3.10	3.80
**Agree**	22.00	3.60	0.62	2.40	3.20	3.60	4.15	4.80
**Assist**	22.00	2.95	0.75	1.60	2.40	2.90	3.55	4.20
**Arrange**	22.00	2.84	0.69	1.40	2.40	3.20	3.40	3.80

Profession: Retired - Summary Statistics

Column	count	mean	std	min	25%	50%	75%	max
**5As Summary Score**	49.00	2.66	0.60	1.40	2.36	2.68	3.04	4.12
**Assess**	49.00	2.28	0.73	1.00	1.80	2.40	2.60	4.20
**Advise**	49.00	2.36	0.79	1.00	1.80	2.20	3.00	4.20
**Agree**	49.00	3.35	0.81	1.00	3.00	3.60	3.80	5.00
**Assist**	49.00	2.66	0.85	1.00	2.00	2.60	3.20	4.60
**Arrange**	49.00	2.63	0.84	1.20	2.00	2.60	3.40	5.00

Profession: Employed - Summary Statistics

Column	count	mean	std	min	25%	50%	75%	max
**5As Summary Score**	42.00	2.79	0.58	1.68	2.48	2.70	3.08	4.28
**Assess**	42.00	2.45	0.86	1.00	1.80	2.40	3.10	4.40
**Advise**	42.00	2.50	0.77	1.00	1.85	2.50	2.95	4.80
**Agree**	42.00	3.39	1.01	1.00	2.80	3.60	4.00	5.00

**Assist**	42.00	3.08	0.81	1.20	2.60	3.20	3.60	5.00
**Arrange**	42.00	2.54	0.73	1.60	1.80	2.60	3.10	4.20

Profession: Unemployed - Summary Statistics

Column	count	mean	std	min	25%	50%	75%	max
**5As Summary Score**	12.00	2.91	0.69	1.60	2.45	2.92	3.39	4.04
**Assess**	12.00	2.33	0.67	1.60	1.80	2.00	2.90	3.40
**Advise**	12.00	2.72	0.90	1.80	1.80	2.50	3.50	4.20
**Agree**	12.00	3.55	1.08	1.00	3.10	3.70	4.20	5.00
**Assist**	12.00	3.07	0.99	1.60	2.35	2.80	3.60	5.00
**Arrange**	12.00	2.87	0.69	1.40	2.60	2.80	3.25	4.20
