# Supplementary material for: Impact of the COVID-19 Pandemic on Lifestyle Behavior and Clinical Care Pathway Management in Type 2 Diabetes: A Retrospective Cross-Sectional Study
Source: Medicina (Kaunas). 2024 Oct 4;60(10):1624. doi: 10.3390/medicina60101624 (PMC11509258; doi:10.3390/medicina60101624)
Supplement: Supplementary file 1 [file medicina-60-01624-s001.zip › Supplementary File S5.pdf]

Supplementary File S5

Inferential Statistics Results PACIC Questionnaire

Comparison	Variable	Test	Statistic	P-value
**M vs F**	5As Summary Score	t-test	-2.4628091092392452	0.015477046840196134
**M vs F**	Assess	Mann-Whitney U test	882.5	0.12125403143060252
**M vs F**	Advise	Mann-Whitney U test	845.5	0.06946088352850179
**M vs F**	Agree	Mann-Whitney U test	911.0	0.18116470398828965
**M vs F**	Assist	t-test	-1.6807675817176333	0.09589827421676263
**M vs F**	Arrange	Mann-Whitney U test	781.5	0.022348268652098303
**More Educated vs Less Educated**	5As Summary Score	t-test	2.2700643737407673	0.029861548570522874
**More Educated vs Less Educated**	Assess	t-test	2.0642008287231333	0.04693258141297781
**More Educated vs Less Educated**	Advise	t-test	1.693399719869066	0.09980019928893426
**More Educated vs Less Educated**	Agree	Mann-Whitney U test	111.5	0.08734866150725355
**More Educated vs Less Educated**	Assist	t-test	1.0699990463470843	0.2923838779491236
**More Educated vs Less Educated**	Arrange	t-test	1.2753867824721359	0.21107802502698103
**Few Members vs More Members**	5As Summary Score	t-test	-0.459361025054207	0.6469621035491031

**Few Members vs More Members**	Assess	Mann-Whitney U test	590.0	0.964107833861137
**Few Members vs More Members**	0.17926951957039605	Mann-Whitney U test	617.0	0.7533642299161432
**Few Members vs More Members**	Agree	Mann-Whitney U test	686.0	0.3163653676146757
**Few Members vs More Members**	Assist	t-test	-1.1672231245214952	0.24586859584164747
**Few Members vs More Members**	Arrange	Mann-Whitney U test	450.0	0.7644443245667789

Inferential Statistics Results Medi Lite Questionnaire

Comparison	Variable	Test	Statistic	P-value
**M vs F**	Fruit	Mann-Whitney U test	1224.0	0.0551017777564224
**M vs F**	Vegetables	Mann-Whitney U test	976.0	0.22800587418381635
**M vs F**	Legumes	Mann-Whitney U test	1225.0	0.2856138367935067
**M vs F**	Cereals	Mann-Whitney U test	1050.0	0.2674567729028776
**M vs F**	Fish	Mann-Whitney U test	1236.0	0.1853588906863619
**M vs F**	Meat and meat products	Mann-Whitney U test	1132.5	0.7557815282067947
**M vs F**	Dairy products	Mann-Whitney U test	1339.0	0.055101756273815285
**M vs F**	Alcohol	Mann-Whitney U test	1391.0	0.01490228796194319
**M vs F**	Olive oil	Mann-Whitney U test	1103.0	0.9155013629169384
**M vs F**	Total	t-test	1.6228112217901856	0.10774713444019582

**More Educated vs Less Educated**	Fruit	Mann-Whitney U test	304.0	0.428936223999666
**More Educated vs Less Educated**	Vegetables	Mann-Whitney U test	269.0	0.6132700126931647
**More Educated vs Less Educated**	Legumes	Mann-Whitney U test	321.0	0.1881410559335147
**More Educated vs Less Educated**	Cereals	Mann-Whitney U test	201.0	0.021922074847871058
**More Educated vs Less Educated**	Fish	Mann-Whitney U test	141.5	0.04010635426499379
**More Educated vs Less Educated**	Meat and meat products	Mann-Whitney U test	251.5	0.9150845444560498
**More Educated vs Less Educated**	Dairy products	Mann-Whitney U test	293.0	0.4289362236621842
**More Educated vs Less Educated**	Alcohol	Mann-Whitney U test	126.5	0.03983779664523114
**More Educated vs Less Educated**	Olive oil	Mann-Whitney U test	270.0	0.4637141143422644
**More Educated vs Less Educated**	Total	Mann-Whitney U test	281.5	0.5760517202471229
**Few Members vs More Members**	Fruit	Mann-Whitney U test	606.5	0.7387423777865842
**Few Members vs More Members**	Vegetables	Mann-Whitney U test	700.0	0.11101880843889372
**Few Members vs More Members**	Legumes	Mann-Whitney U test	567.0	0.8435012337175671
**Few Members vs More Members**	Cereals	Mann-Whitney U test	604.5	0.5171247547264602
**Few Members vs More Members**	Fish	Mann-Whitney U test	589.5	0.9588575035785997
**Few Members vs More Members**	Dairy products	Mann-Whitney U test	614.5	0.7387423868865952
**Few Members vs More Members**	VERDURA	Mann-Whitney U test	473.5	0.2316029691614444
**Few Members vs More Members**	Alcohol	Mann-Whitney U test	569.0	0.861295162836137
**Few Members vs More Members**	Olive oil	Mann-Whitney U test	441.0	0.005479006983786122

**Few Members vs More Members**	Total	Mann-Whitney U test	177.5	4.294376589055955e-05
